# Nutrition literacy status and its association with adherence to the Mediterranean diet, anthropometric parameters and lifestyle behaviours among early adolescents

**DOI:** 10.1017/S1368980023001830

**Published:** 2023-10

**Authors:** Gamze Yurtdaş Depboylu, Gülşah Kaner, Melisa Süer, Mesude Kanyılmaz, Duygu Alpan

**Affiliations:** 1 Izmir Katip Celebi University, Faculty of Health Sciences, Departments of Nutrition and Dietetics, İzmir 35620, Turkey; 2 Department of Science Education, Çiğli Akiş Öğütçü Secondary School, İzmir, Turkey; 3 Department of English Language Teaching, Çiğli Akiş Öğütçü Secondary School, İzmir, Turkey

**Keywords:** Nutrition literacy, Early adolescent, Mediterranean diet, Lifestyle behaviours

## Abstract

**Objective:**

To evaluate nutrition literacy status and its association with adherence to the Mediterranean diet (MD), anthropometric parameters and lifestyle behaviours among early adolescents.

**Design:**

This is a cross-sectional study. Nutrition literacy was evaluated using the ‘Adolescent Nutrition Literacy Scale’. Dietary intake was assessed by 24-h food recall. The ‘Mediterranean Diet Quality Index’ was used to evaluate adolescents’ adherence to the MD. Physical activity was assessed using the International Physical Activity Questionnaire (IPAQ). Body weight, height, waist, hip and neck circumference were measured.

**Setting:**

Four secondary schools in İzmir, Türkiye

**Participants:**

The study included 1074 secondary school students.

**Results:**

Adolescents’ nutrition literacy was at a moderate level. Nutrition literacy scores were significantly lower in those who skip main meals. Adolescents with high nutrition literacy had higher intakes of fibre, protein, protein, Ca, K, Mg, P, vitamin C, folate and Fe intake than those with low and moderate nutrition literacy (*P* < 0·05). According to IPAQ, active adolescents had higher nutrition literacy scores than inactive adolescents. There was no significant difference in BMI and anthropometric measurements of the adolescents according to their nutrition literacy level. Linear regression analysis showed that each unit increase in nutrition literacy increased adherence to the MD by 0·286 points (*β* = 0·286) and decreased total screen time by 0·182 points (*β* = –0·182).

**Conclusions:**

These findings showed that nutrition literacy among early adolescents was not optimal, and a higher nutrition literacy score was significantly associated with higher MD adherence, and healthy eating habits and lifestyle behaviours.

Adolescence is considered the best period to develop throughout life positive health behaviours. Lifelong eating habits, which are a part of the lifestyle, are shaped in this period^(1)^. Recently, it has been reported that adolescents are gradually moving away from the Mediterranean diet (MD) (based on plant-based food, MUFA, complex carbohydrates, and decreased intake of red and processed meats) which represents a healthy and sustainable diet for all age groups and has a significant impact on the prevention of cardiovascular and metabolic disorders^(2–4)^. Numerous studies have shown that adolescents have low adherence to the MD, but high adherence to the Western diet pattern, which is characterised by a high-energy diet style and ultra-processed foods, rich in saturated fats and low in micronutrients^(4–6)^. In addition, it was reported that 80 % of adolescents lack physical activity, and screen-based sedentary behaviours such as watching TV and playing video games are very common among adolescents^(7,8)^. Unhealthy eating habits, sedentary behaviours and physical inactivity are all recognised as risk factors for chronic diseases, including diabetes mellitus, CVD and obesity^(9,10)^. Recently, it has been reported that nutrition-related problems and behaviours among children and adolescents are associated with nutrition literacy^(1,9,11)^.

Nutrition literacy is defined as ‘the degree to which individuals have the ability to receive, process, and understand the nutritional knowledge and skills necessary for making appropriate nutrition decisions’^(12)^. A cross-sectional study involving 2·869 adults has shown a significant association between nutrition literacy and adherence to the MD^(13)^. Tehrani et al.^(14)^ found that a higher nutrition knowledge score was associated with a higher MD adherence score in Iranian female adolescents. A study conducted on adolescents found that there was a relationship between nutrition literacy and BMI, daily lifestyle behaviours, and eating habits^(1)^. Another study found that nutrition literacy scores were positively related to smaller fast-food portion sizes and lower frequency of intake of packaged or processed snacks among school age children and adolescents^(15)^.

Determining nutrition literacy status and understanding the determinants of healthy eating and lifestyle behaviours can help adopt effective strategies to promote health in early adolescents^(15,16)^. There is limited data regarding the influence of nutrition literacy on MD and lifestyle behaviours. Moreover, to the best of our knowledge, there is no study evaluating nutrition literacy status among Turkish secondary school students and the relationship between nutrition literacy, physical activity level, total screen time, and anthropometric measurements (waist, hip and neck circumference). Therefore, the main purpose of this study was to determine the nutrition literacy status of early adolescents and its association with adherence to the MD, anthropometric measurements, and lifestyle behaviours, including eating habits, dietary intake, physical activity level and screen time.

## Materials and methods

### Design and participants

This cross-sectional study was carried out from December 2021 to April 2022 in public secondary schools in the Çiğli district in İzmir (a city west of Türkiye). Schools were selected using stratified sampling. To reflect the entire Çiğli district, Çiğli was divided into four different regions: north, south, east and west. The total number of public secondary schools in this district was 38, and the number of public secondary schools in each strata was approximately similar to each other. One school from each strata was determined by randomisation. Four public secondary schools were included in this study. For secondary schools (intermediate education) in Turkiye, the duration of education is 4 years and covers grades 5–8. This education is mandatory for all citizens and free at public schools. Schools included in this study are half day-time schools with a canteen, an outdoor football field and an indoor sports hall. These schools do not receive school meal support from the government. Nutrition literacy education and nutrition literacy-enhancing activities are not available at these schools.

**Fig. 1:**
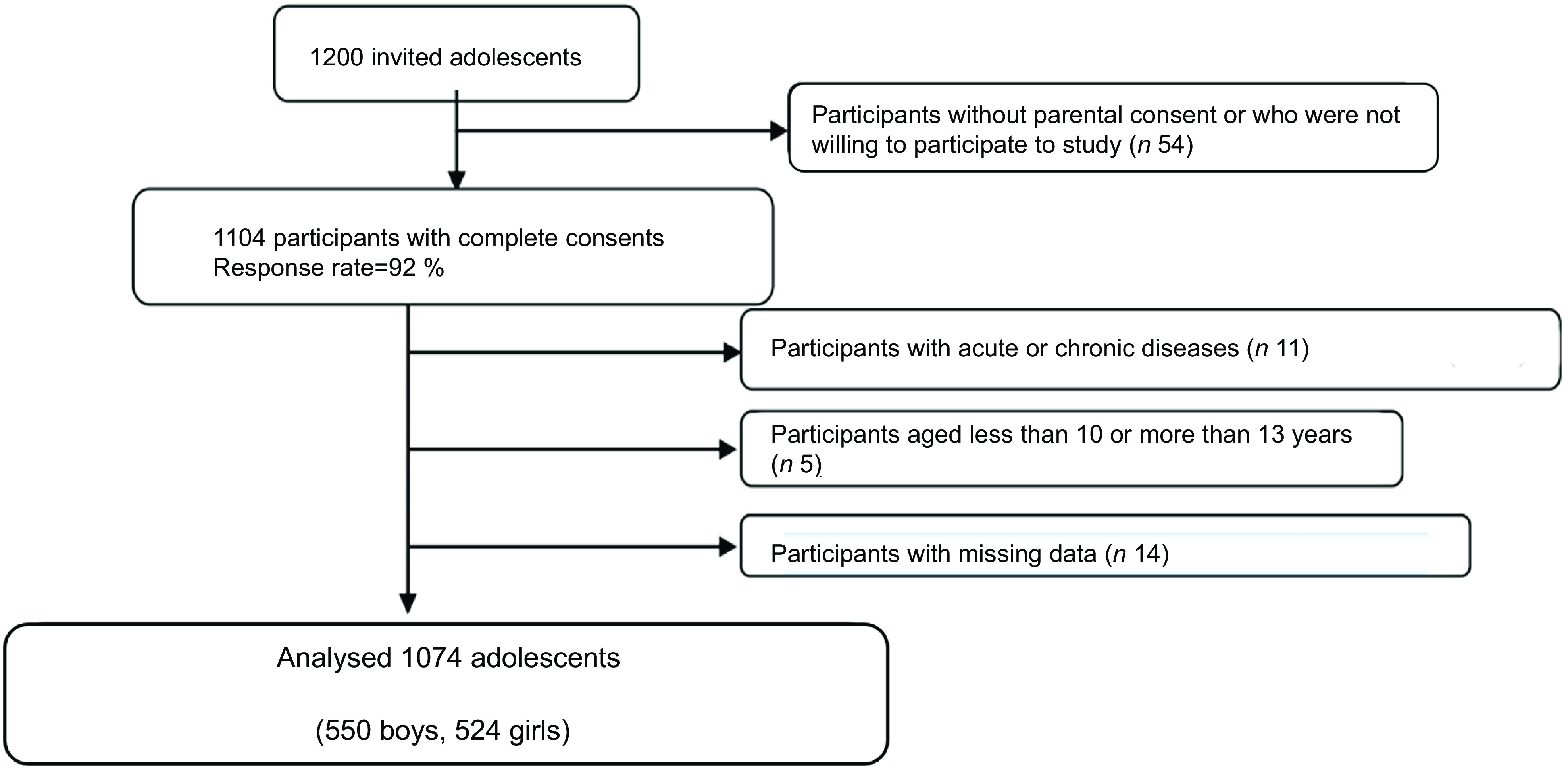
Flow chart of participant recruitment

After obtaining the approval of the ethics committee, necessary permission was obtained from the Provincial Directorate of National Education to conduct the study in these schools. All students from the same school and classroom were recruited if they met the inclusion criteria and provided oral consent to participate. The study sample included 1074 secondary school students of 550 boys and 524 girls (aged 10–13 years) (response rate, 92 %). Inclusion criteria were willingness to participate and being aged between 10 and 13 years. Participants were excluded based on the following criteria: aged less than 10 or more than 13 years, and having severe acute or chronic diseases. The flow chart of participant recruitment is presented in Fig. 1.

### Data collection

After the necessary permissions were obtained, the researchers visited all the schools and informed the students about the study. The written informed consent forms were delivered to the parents by volunteer students. The next day, parents signed the informed written consent form and sent it back to the researchers. The data were collected in the classroom using face-to-face interview techniques after consent forms were received. To prevent mistakes and misunderstandings, the researchers read and clarified the questionnaire to the students prior to their completion. After that, completed questionnaires were collected from the students.

The questionnaire was divided into five sections. The first part of the questionnaire asked for information about the students’ sociodemographic data (age, gender, grade, parent’s employment status and education level, etc.), and dietary habits (generally preferred foods for snacks, main meals, snacks pattern, frequency of fast-food consumption and daily water consumption) and physical activity status (duration of sleep and screen time). The second section consisted of the “Adolescent Nutrition Literacy Scale” to evaluate the nutritional literacy levels of adolescents. The third section incorporated Mediterranean Diet Quality Index for Children and Adolescents (KIDMED) to assess adherence to the MD. The fourth section assessed physical activity levels using the International Physical Activity Questionnaire (IPAQ). The 24-h dietary recall made up the fifth and final section.

### Mediterranean Diet Quality Index (KIDMED)

The KIDMED questionnaire was applied to measure adolescents’ adherence to the MD. KIDMED, developed by Serra-Majem et al.^(17)^, is an index consisting of a total of sixteen statements that include the characteristics of the MD. It was developed to measure dietary adequacy between the ages of 2–24 years. Of the expressions included in the KIDMED index, twelve are positive and four are negative expressions, and those who answer yes to positive expressions get +1 and those who answer yes to negative expressions get –1 points. According to the total scores of the adolescents from the index, adherence to the MD was interpreted by dividing it into three categories. These are (1) low adherence (0–3 points), (2) moderate adherence (4–7 points) and (3) high adherence (8–12 points)^(17)^.

### Adolescent Nutrition Literacy Scale

‘Adolescent Nutrition Literacy Scale (ANLS)’ was used to determine the nutrition literacy status of adolescents. This scale was developed by Bari^(18)^ and was adapted into Turkish by Türkmen et al.^(19)^ ANLS consists of twenty-two items and each item is on a five-point scale, with scores ranging from 1 to 5 (1 = strongly disagree, 2 = disagree, 3 = undecided, 4 = agree and 5 = strongly agree). The minimum score that can be obtained from this scale is 22, and the maximum score is 110^(19)^. A score of 22–57·2 indicates ‘low’, a score of 57·2–74·8 is considered ‘moderate’ and a score of 74·8–110 is classified as ‘high nutrition literacy’^(20)^.

### Physical activity

IPAQ was used to evaluate the physical activity of participants. The validity and reliability of the questionnaire in Turkey were performed by Öztürk^(21)^. This brief form has seven questions and asks about time spent standing, walking, doing moderately intense activities and doing vigorous activities. According to their level of physical activity, adolescents were divided into three categories: ‘inactive’, ‘moderately active’ and ‘active’^(21)^.

### Dietary intake

Dietary intake was assessed by the food consumption record (24-h dietary recall). To verify that adolescents accurately indicated the amount of food they ingested, the ‘Food and Nutrient Photo Catalogue’ was used. The food consumption record was completed by contacting the parents of the students who could not remember or remember incompletely what they ate the previous day. BeBiS (Ebispro for Windows, Germany; Turkish Version/BeBiS 8) was used for analysing dietary energy and nutrients.

### Anthropometric measurements

Weight was measured with minimum clothing, without shoes, using a digital scale (Tanita BC-532). A handheld stadiometer with 0·1 cm precision was used to measure height while standing with feet close together and the head in the Frankfort plane. Waist circumference was assessed using a non-flexible measuring tape at the end of expiration from the midpoint between the lowest rib and the crista iliac. Hip circumference was measured from the highest circumference of the hip at the back, standing on the side of the participants. Neck circumference was measured just below the larynx, with the head in the Frankford plane. BMI was calculated as weight (kg)/height (m^2^). The adolescents were divided into four groups based on their BMI-for-age percentiles: ‘underweight’ (< 5 percentile); ‘normal’ (≥ 5–< 85 percentile); ‘overweight’ (≥ 85–< 95 percentile); and ‘obese’ (≥ 95 percentile)^(22)^.

### Statistical analyses

Data were analysed using SPSS 23.0 (SPSS Inc.). The normality test was performed by the Kolmogorov–Smirnov test. Numbers (*n*) and percentages (%) were used to present categorical data; normally distributed data were represented by the mean and standard deviation and non-normally distributed data by the median and interquartile range. The Student’s *t* test and one-way ANOVA were used to compare the descriptive characteristics of participants with their mean ANLS scores. In multiple comparisons of these variables, the ‘Tukey’ test was used when the variances were equal and the ‘Tamhane T2’ test was used if not equal. ‘Kruskal–Wallis *H* test’ was used to compare the dietary intake and anthropometric measurements data that did not fit the normal distribution according to the nutritional literacy level, and one way ANOVA was used for the normally distributed data. Multivariable linear regression analysis was carried out to determine the association between nutrition literacy and some related factors. A two-sided *P* value of < 0·05 was considered to be statistically significant.

## Results

The general characteristics of participants are presented in Table 1. Boys and girls were almost equivalent in number (boys, 51·1 %), and the mean age was 11·9 ± 1·13 years. 29·1 % of adolescents were in seventh grade, and 60·1 % of family income was equal to their expenses. The education level of the parents was mostly high school. According to BMI for age, 44·9 % of students had normal BMI values; on the other hand, 24·4 % were classified as obese. While there was no significant difference between girls and boys in terms of grade, parents’ education and occupation, and income level, there was a difference in terms of BMI distribution. The mean nutrition literacy score of adolescents was 68·4 ± 8·98 (moderate level). There were no significant differences between boys and girls scores in nutrition literacy (*P* > 0·05).

**Table 1:** Characteristics of study participants

		*n* (%) or Mean ± sd						
		Boys (*n* 550)		Girls (*n* 524)		Total (*n* 1074)		
		*n*	%	*n*	%	*n*	%	*P* value
Grade	5th	135	24·5	137	26·1	272	25·3	0·617
	6th	130	23·6	107	20·4	237	22·1	
	7th	160	29·1	153	29·2	313	29·1	
	8th	125	22·7	127	24·2	252	23·5	
Father education	Iliterate	39	7·1	27	5·2	66	6·1	0·064
	Literate	12	2·2	17	3·2	29	2·7	
	Primary school	98	17·8	119	22·7	217	20·2	
	Secondary school	104	18·9	117	22·4	221	20·6	
	High school	208	37·8	171	32·6	379	35·3	
	University	89	16·2	73	13·9	162	15·1	
Mother education	Iliterate	43	7·8	33	6·3	76	7·1	0·315
	Literate	27	4·9	31	5·9	58	5·4	
	Primary school	126	22·9	141	26·9	267	24·9	
	Secondary school	105	19·1	112	21·4	217	20·2	
	High school	176	32	145	27·7	321	29·8	
	University	73	13·3	62	11·8	135	12·6	
Mother employment status	Unemployed	20	3·6	19	3·6	39	3·6	0·116
	Housewife	352	64	349	66·6	701	65·3	
	Retired	4	0·7	7	1·3	11	1·1	
	Officer	19	3·5	19	3·6	38	3·5	
	Labour	96	17·5	59	11·3	155	14·4	
	Self-employment	23	4·2	29	5·5	52	4·8	
	Other	36	6·5	42	8·1	78	7·3	
Father employment status	Unemployed	16	2·9	13	2·5	29	2·7	0·622
	Retired	39	7·1	28	5·3	67	6·2	
	Officer	44	8·1	40	7·6	84	7·8	
	Labour	245	44·5	221	42·2	466	43·4	
	Self-employment	109	19·8	117	22·3	226	21·1	
	Other	97	17·6	105	20·1	202	18·8	
Income level	Income is less than expense	20	3·6	15	2·9	35	3·3	0·551
	Income is equal to expense	336	61·1	310	59·2	646	60·1	
	Income is more than expense	194	35·3	199	38·0	393	36·6	
BMI	Underweight	74	13·5	64	12·2	138	12·8	**<0·001**
	Normal	209	38·0	273	52·1	482	44·9	
	Overweight	104	18·9	88	16·8	192	17·9	
	Obese	163	29·6	99	18·9	262	24·4	
Adolescent Nutrition Literacy Scale score*								
Mean		67·9		68·9		68·4		0·105
** ** sd		8·92		9·03		8·98		

Significant values are shown in bold (*P* < 0·05).

* Data are expressed as mean ± sd.

According to IPAQ, active adolescents had higher nutrition literacy scores than inactive (sedentary) students (*P* < 0·05). Adolescents who stated that they spent less than 1 h or 1–2 h per d in front of the screen had a higher nutrition literacy score than those who stated that they spent 3three h or more than 3 h. Besides, nutrition literacy scores were significantly lower in those who skip main meals (*P* < 0·05). Nutrition literacy scores of adolescents who preferred milk/yogurt and fruits for snacks were higher than those who preferred sweet foods (*P* < 0·05). Those who did not consume fast food had a higher score on nutrition literacy than those who did, both daily and 4–5 d a week. The mean nutrition literacy of those with high adherence to the MD was higher than those with moderate and low adherence (*P* < 0·05). Adolescents who consumed more than 6–8 glasses of water per d had a higher nutrition literacy score than those who consumed 3–6 glasses of water daily (Table 2).

**Table 2:** Mean score of nutrition literacy according to some possible determinant factors

Variables		*n*	%	Mean	sd	P* value
Physical activity level	Inactive^a^	200	18·6	66·8	8·23	**0·014**
	Moderately active^b^	426	39·7	68·5	8·96	c > a
	Active^c^	448	41·7	69·0	9·26	
Total screen time**	0–1 h^a^	36	3·4	71·4	11·52	**< 0·001**
	1–2 h^b^	590	54·9	69·9	8·74	a > c; b > c
	3 h and more^c^	448	41·7	66·2	8·63	
Duration of sleep	0–6 h	160	14·9	67·9	8·59	0·112
	6–8 h	625	58·2	68·1	9·21	
	9–12 h	289	26·9	69·3	8·66	
Height for age	3–15 percentile	101	9·4	67·3	9·62	0·335
	15–85 percentile	661	61·5	68·3	9·23	
	85–95 percentile	144	13·4	68·6	7·92	
	≥ 95 percentile	168	15·6	69·3	8·43	
BMI for age	Underweight	138	12·8	68·2	8·91	0·883
	Normal	482	44·9	68·4	9·10	
	Overweight	192	17·9	69·0	9·11	
	Obese	262	24·4	68·2	8·75	
Skipping main meals during the day	Yes	603	56·1	67·3	8·60	**< 0·001**
	No	471	43·9	69·8	9·27	
Skipped main meal	Breakfast	294	48·8	67·6	8·66	0·262
	Lunch	259	43·0	66·7	8·45	
	Dinner	50	8·3	68·6	8·94	
Foods preferred for snacks	Milk/yogurt ^a^	139	13·7	69·7	8·52	**< 0·001**
	Nuts (hazelnuts, Peanuts, walnuts, etc.)^b^	123	12·2	69·4	8·43	
	Fruits ^c^	346	34·2	69·7	9·34	b > e; a,c > d
	Sweet foods (cakes, cookies, chocolate, etc.)^d^	254	25·1	66·5	8·71	
	Chips, crackers and popcorn^e^	115	11·4	66·5	8·62	
	Carbonated drinks^f^	34	3·4	66·7	9·38	
Water consumption	3–< 6 glasses^a^	432	40·2	67·3	8·59	**0·004**
	≥ 6–< 8 glasses^b^	291	27·1	69·3	9·37	c > a; b > a
	≥ 8 glasses^c^	351	32·7	69·0	9·01	
Frequency of fast-food consumption	Never ^a^	174	16·2	69·7	9·81	**< 0·001**
	1 d per week ^b^	527	49·1	68·6	8·79	a > e,d, c
	2–3 d per week ^c^	270	25·1	68·7	8·64	
	4–5 d per week ^d^	61	5·7	65·5	7·82	
	Everyday ^e^	42	3·9	63·3	9·36	
KIDMED index score	≤ 3 (low adherence)ª	235	21·9	65·0	8·08	**< 0·001**
	4–7 (moderate adherence)^b^	601	56·0	68·3	8·75	c > b,a; b > a
	≥ 8 (high adherence)^c^	238	22·1	71·1	9·02	

Significant values are shown in bold (*P* < 0·05).

* Statistical significance of means difference was examined using one-way ANOVA.

** Total screen time (daily computer, tablet, smartphone and TV usage time). Each variable was identified with a different letter (a, b, c, d, e, and f).

Dietary intake and anthropometric measurements according to nutrition literacy level are shown in Table 3. Adolescents with high nutrition literacy had higher intakes of fibre (g), protein (g), protein (%), Ca (mg), K (mg), Mg (mg), P (mg), vitamin C (mg), folate (mcg) and Fe (mg) intake than those with low and moderate nutrition literacy (*P* < 0·05). There was no significant difference in the energy, carbohydrate, fat intake and anthropometric measurements of the adolescents according to their nutrition literacy level.

**Table 3:** Dietary intake and anthropometric measurements according to nutrition literacy level

Variables	Low nutrition literacy (*n* 104)		Moderate nutrition literacy (*n* 718)		High nutrition literacy (*n* 252)		*P* value
Dietary intake (Median (IQR) or Mean ± sd)							
	Mean	sd	Mean	sd	Mean	sd	
Total energy intake (kcal)	1462·1	575·72	1497·1	546·85	1552·3	538·24	0·265
Protein (g)*	46·0	29·98^a^	51·1	26·33^a^	56·4	28·97^b^	**0·002**
Protein (% of energy)	14·5	5·07^a^	15·0	4·08^a^	15·7	3·89^b^	**0·018**
Carbohydrate (g)	170·5	75·04	176·2	76·95	174·0	70·60	0·741
Carbohydrate (%)	47·3	9·97	48·1	11·37	46·2	10·70	0·061
Total fat (g)	62·5	31·56	62·2	30·51	66·8	32·19	0·129
Total fat (% of energy)	38·0	9·15	36·7	10·60	37·9	10·16	0·183
Fibre (g)*	12·2	8·53^a^	13·9	9·36^a^	15·2	11·40^b^	**0·002**
Cholesterol (mg)*	153·2	291·73	218·9	271·81	238·7	269·18	0·150
Ca (mg)*	588·7	370·95^a^	607·5	405·14^a^	700·0	454·80^b^	**0·001**
Mg (mg)	204·9	99·21^a^	210·1	85·27^a^	230·5	99·41^b^	**0·005**
P (mg)	870·7	399·39^a^	905·2	351·9^a^	1004·5	395·06^b^	**< 0·001**
K (mg)	1744·3	759·66^a^	1832·5	782·0^a^	2032·0	842·28^b^	**0·001**
Na (mg)*	2195·5	1834·57	2442·8	1727·44	2471·5	1351·59	0·314
Vitamin C (mg)*	44·5	54·67^a^	61·5	86·59^b^	68·2	97·38^b^	**< 0·001**
Vitamin E (mg)*	6·7	7·06	7·0	7·04	8·2	7·31	0·484
Folate (mcg)*	149·6	119·09^a^	176·5	123·49^b^	208·9	151·54^c^	**< 0·001**
Fe (mg)*	6·2	4·39^a^	6·6	3·99^a^	6·9	5·07^b^	**0·001**
Zn (mg)*	6·3	5·15	6·9	3·74^a^	7·5	4·19^b^	**0·019**
Anthropomentric measurements (Mean ± sd)							
Body weight (kg)	48·9	14·89	50·0	14·01	49·1	13·75	0·565
Height (cm)	153·6	10·02	155·0	10·25	153·5	9·61	0·101
BMI (kg/m^2^)	20·5	4·69	20·6	4·46	20·5	4·36	0·945
Waist circumference (cm)	71·8	12·81	71·5	11·34	71·6	11·14	0·969
Hip circumference (cm)	85·8	10·85	86·5	10·65	86·1	11·05	0·741
Neck circumference (cm)	29·6	3·22	29·7	3·06	29·5	3·05	0·550

Significant values are shown in bold (*P* < 0·05).

* Data are expressed as Median (IQR).

Dissimilar values (superscripts a, b and c) of each row are significantly different.

To assess the effect of nutrition literacy on KIDMED score (adherence to the MD) and total screen time by controlling for potentially confounding factors (age, gender, income level and parent’s education), multiple linear regression analysis was conducted. According to the results of the regression analysis, when the significance level corresponding to the F value was taken into account, model 1 and model 2 established were statistically significant (F = 28·682; *P* < 0·05 for model 1; F = 18·700; *P* < 0·05 for model 2) (Table 4). In the first model, nutrition literacy explained 13·4% of the variance in the KIDMED score of adolescents (adjusted R² = 0·134), while each unit increase in nutrition literacy increased KIDMED 0·286 points (*β* = 0·286). In the second model, while nutrition literacy influenced 9·0 % of adolescents’ total screen time (adjusted R² = 0·090), each unit increase in nutrition literacy decreased total screen time 0·182 points (*β* = –0·182). There were no autocorrelation problem in the established models. Durbin Watson’s values for each model were between 1·5 and 2·5.

**Table 4:** The effect of nutrition literacy on KIDMED and total screen time

	Model 1						Model 2					
	KIDMED score*						Total screen time*					
	Standardised coefficients *β*	*P* value	95 % Cl	Adjusted R²	F	Model (p)	Standardised coefficients *β*	*P* value	95 % Cl	Adjusted R²	F	Model (p)
Nutrition literacy score**	0·286	**< 0·001**	0·064, 0·095	0·134	28·682	**< 0·001**	−0·182	**< 0·001**	−0·015, –0·008	0·090	18·700	**< 0·001**
Durbin Watson (1·5–2·5)	2·002						2·084					

All models adjusted for gender, age and income level and parent’s education. Significant values are shown in bold (*P* < 0·05).

* Dependent variable.

** Independent variable.

## Discussion

This study’s findings demonstrated that the nutrition literacy of secondary school students in Turkey was at a moderate level and higher nutrition literacy was associated with several lifestyle and dietary pattern outcomes including higher MD adherence, higher water and lower fast-food consumption, and lower total screen time. In addition, this study showed that there was no relationship between nutrition literacy and BMI and anthropometric measurements.

Evaluation of nutrition literacy status among secondary school children can help in improving nutritional health and implementing useful solutions^(16)^.This study revealed that the nutrition literacy of Turkish secondary school students was at a moderate level. This result is consistent with Liu et al.^(23)^ study and Zeng et al’s^(24)^ study conducted with middle school children^(24)^. Similarly, Turkish high school students’ nutritional literacy was found to be moderate^(25,26)^. The moderate nutrition literacy determined among early adolescents indicates that the nutritional literacy status of Turkish secondary school students should be improved, and this result gives key messages to educators and public healthcare planners to have more consideration for food and nutrition-related knowledge and develop new public health strategies focus on an increasing level of nutrition literacy of secondary school children.

In the present study, nutrition literacy was associated with healthy eating habits including higher water and lower fast-food consumption and consuming milk/yogurt and fruits for snacks. Moreover, nutrition literacy scores were higher in adolescents who do not skip main meals. Consistent with these results, previous studies have reported that the eating habits of adolescents change positively with the increase in their nutrition literacy^(1,15)^. A recent study found that among school age children and adolescents, nutrition literacy scores were positively related to smaller fast-food portion sizes and lower frequency of intake of packaged or processed snacks^(15)^. Another study demonstrated that the nutrition literacy of students who consume fast food once a week was higher than those who consume it every day^(27)^. Furthermore, high nutrition literacy was found associated with frequencies of main meal consumption^(28)^ and increased daily water consumption^(29)^. Taken together, these findings suggest that nutrition literacy may play an important role in children’s eating habits and high nutrition literacy may enable adolescents to make healthy diet choices.

Diet quality is associated with healthy eating habits and is an important nutritional factor that improves the quality of life^(30)^. Improvement in diet quality in adolescents has positive effects such as a decrease in obesity indicators, an increase in cognitive functions and an improvement in mental health^(31)^. This study showed that higher adherence to the MD (higher diet quality) was associated with higher nutrition literacy scores among early adolescents. This finding is consistent with the results of previous studies. Taylor et al.^(11)^ reported that high nutrition literacy may enable individuals to adherence a high-quality prudent diet or MD in adults. In a study conducted with Iranian female adolescents, higher nutrition knowledge was significantly associated with a higher Mediterranean dietary pattern adherence score^(14)^. In a study on adults in Italy, a significant association was demonstrated between nutrition knowledge and adherence to the MD^(13)^. Similarly, Wall et al.^(32)^ revealed that higher nutrition literacy was associated with a healthier dietary eating pattern among adults. The association between MD adherence and nutrition literacy suggests that nutrition literacy is an important predictor of adherence to the MD among early adolescents.

This study demonstrated that adolescents with high nutrition literacy had higher intakes of fibre, protein, protein (%), Ca, K, Mg, P, vitamin C, folate and Fe intake than those with low and moderate nutrition literacy. Similarly, a significant positive relationship was found between nutrition literacy and dietary protein (%), fibre, and K intake among women^(33)^. Joulaei et al.^(16)^ reported that increased nutrition literacy was associated with lower sugar intake, improved energy balance in boys and enhanced dairy intake in girls. Another study demonstrated that high nutrition literacy predicted high consumption of low-fat dairy products, vegetables, nuts and seeds, olive oil, and soya products^(11)^. It has been reported that low nutrition literacy status may be a barrier to dietary diversity and nutritional adequacy in school age children^(34)^. Previous studies results have shown that high nutrition literacy is associated with increased fruit and vegetable consumption^(28,35)^. These results indicate that adolescents with high nutrition literacy consume more foods that are sources of protein, fibre, Ca, A and C vitamins (vegetables, fruits, whole grains, legumes and nuts), in accordance with the MD. The MD is rich in vitamins and minerals and contains high levels of complex carbohydrates and fibre^(36)^. In this context, these results support the link between nutrition literacy and MD adherence.

Several studies have shown that sedentary behaviours increase and the level of physical activity decreases among adolescents^(26,37,38)^. It has been reported that adolescents spend their spare time mostly in front of screens such as smartphones, tablets, game consoles and televisions^(39)^. Consistent with the literature in this study, the rate of adolescents who stated that they spent 3 or more hours in front of the screen was found to be high (41·7 %). Relationships between screen time and negative health effects including obesity and inactivity have been well documented^(39)^. This study showed that active adolescents had higher nutrition literacy scores than inactive (sedentary) adolescents. The multiple linear regression analysis revealed that lower total screen time was associated with higher nutrition literacy scores. Supporting this study, the nutrition literacy scores of those who watched TV < 1 h/d were found to be significantly higher than those of the students who spent more time watching TV^(2)^. Consistently, another study revealed that higher nutrition knowledge was significantly associated with good physical activity behaviour in students^(40)^. Additionally, some authors reported that there is a relationship between health literacy, which is a concept that includes nutritional literacy and is defined as individuals’ ability to make decisions that have a positive impact on their own health, and active lifestyle^(41)^. Overall, these results suggest the nutrition literacy predictor of a healthy lifestyle among early adolescents.

There are many studies addressing nutrition literacy and weight status. However, the results of these studies were inconsistent^(2,11,42,43)^. In some studies, nutrition literacy was inversely associated with overweight/obesity among adolescents^(43)^, while in others a positive relationship between nutrition literacy and BMI has been reported^(11)^. This study showed no association between nutrition literacy and BMI, neck, waist, and hip circumference. In line with these results, Taleb and Itani^(8)^ found no association between nutrition literacy and BMI among adolescents. Regarding anthropometric measurements, to the best of our knowledge, there is no study investigating the relationship between nutrition literacy with waist, hip and neck circumference among adolescents. Although nutritional literacy was associated with healthy lifestyle behaviours, the non-significant association between nutrition literacy and BMI and anthropometric measurements can be explained by the fact that these parameters are influenced not only by the nutritional literacy level but also by other factors such as lifestyle, environmental, psychological and genetic factors^(44)^. The present study did not examine the relationship between these other factors and BMI. Further research is required to understand the relationship between nutrition literacy and BMI and anthropometric measurements.

This study has some limitations. First, causal relationships between nutrition literacy and physical activity, diet quality, and intake could not be determined from this study due to its cross-sectional design. Second, the self-reported survey is subject to social desirability and response bias. Third, this study was conducted among secondary school students in Turkey; therefore, these results may not be generalised to other age groups. In spite of these limitations, this study is strengthened by its large sample size, use of validated instruments and robust hypotheses-driven analyses. Moreover, dietary intake was assessed with 24-h dietary recall, which allows analysis of typical dietary intake in adolescents.

### Conclusion

The findings of this study showed that nutrition literacy among early adolescents was not optimal, and a higher nutrition literacy score was significantly associated with higher MD adherence, healthy lifestyle behaviours including healthy eating habits and lower screen time. These results suggest that finding methods to improve early adolescents’ nutrition literacy is crucial for promoting healthy dietary patterns and lifestyle behaviours. Adolescents’ awareness should be increased to develop healthy lifestyle behaviours, and health policies should be developed in this regard by creating appropriate educational content. Future studies with long-term follow-up plans are necessary to comprehend how nutrition literacy affects the behaviours that contribute to healthy lifestyle behaviours. Moreover, future research should concentrate on concepts that can enhance adolescents’ nutrition literacy to build nutritional interventions that will encourage healthy eating among secondary school students.
